# Annotations

**Published:** 1910-04-16

**Authors:** 


### Annotations.

### A Memorial to the late Dr. Savill.

The sad death of the late Dr. Thomas Dixon Savill in Algiers is too recent for us to need to bring it back to the remembrance of our readers. It is proposed by those who knew and appreciated him to raise a memorial to perpetuate his name, and for this purpose to institute a fund. With this proposal his many friends and pupils, who valued his friendship and appreciated his teaching, will be in cordial agreement. The precise form that the memorial will take must depend on the amount collected, but inasmuch as Dr. Savill has done so much to advance the study of nervous diseases, that most intricate of all branches of medicine, it is hoped that sufficient may be subscribed to endow a scholarship for neurological research. This would be in direct continuation of a wish frequently expressed by him. Indeed, Dr. Savill had formulated a scheme to carry out such an ideal shortly before he died. We feel certain, therefore, that the object of this fund will meet with the approval of all those who value his memory as they valued his friendship. Any contribution that readers may wish to make should be sent to the Treasurer, Savill Memorial Fund, 60 Upper Berkeley Street, W., and crossed the London County and Westminster Bank, 1 Connaught Street, Hyde Park, W. The trustees of the fund are Dr. Harry Campbell, Mr. J. H. Chaldecott, and Dr. George Davis.

### Medical Defence.

Few annual reports are so interesting in themselves as those issued by the Medical Defence Associations of the type of the Medical Defence Union and the London and Counties Medical Protection Society, Ltd., associations which every medical man should join the moment his name has been handed in for official registration. The importance of the work carried on by these societies needs no explanatory comment from us; anyone who wishes to see details about that work, which is carried on not only in the interests of individual members but in the interests of the whole profession, should apply to the general secretaries for copies of their annual reports and read the extended information given concerning the activities of the organisations during the past twelve months. These societies have helped many a member out of difficulties which were placed in his way through no fault of his own. By really interesting themselves in questions of ethical importance they have done much good by pointing out where an error has been committed and by taking steps to repair any indiscretion which a member may inadvertently have been guilty of. Their merit is enhanced by the fact that they do such work perseveringly, quietly, and steadily, making no advertisement and looking for no reward beyond the gratitude of the profession. Perhaps for that very reason, on account of their modesty, such associations are insufficiently well known to the younger practitioners, and we venture to think that it is the duty of every member and of everyone who knows about their work and its value, to bring their objects to the notice of the newly qualified. An excellent way of doing so would be to forward copies of the annual reports. These reports, giving as they do details of the work done, are highly interesting; they contain many valuable hints that will be of use to new men, and are usually most readable productions, the reading of which is bound to bring profit and instruction.

### Five per Cent. Philanthropy.

According to a paragraph which has this week appeared in the daily press, a scheme is on foot for the establishment of a national dental organisation for the benefit of those who are willing to pay for dental treatment and appliances, but are unable to afford the ordinary fees of dentists. Full details of the scheme are not yet available, but a preliminary announcement has been made. An anonymous donor has offered a sum of £200,000 as capital for the enterprise, which is described as philanthropy on a 5 per cent. basis, and this sum is put at the disposal of Mr. Rayner, the Secretary of the British Dental Association, for the purpose of starting a clinic in London; other clinics, to the number of 500, are contemplated all over the kingdom if the scheme proves a success. The offer has not yet been accepted, and the British Dental Association will naturally have to give very close attention both to the principles and the details of the proposed organisation. Mr. Rayner, in fact, has told an interviewer that the offer cannot be accepted unless he is assured of the support of the medical and dental professions and of the many societies already in existence. The scheme, he believes, is honestly designed to meet a public need; there are many who cannot and will not accept hospital treatment and yet would be inconvenienced by the fees of a good dentist, and this class often does without dental aid and suffers accordingly. It is said that all work done will be remunerated at moderate but sufficient rates, the capital put into it will bear interest, and if people desire they can pay their bills by weekly instalments. Further, an educative campaign will be started, with the object of persuading the public to submit to conservative treatment instead of waiting until the mouth contains nothing but septic stumps which must perforce be extracted. One of the best arguments in favour of the proposal is that it will relieve the hospitals of a certain class who have really no proper right to use them, but who at present can hardly be refused. The industrious and often intelligent artisan (and his family), who may be earning wages of 30s. to 50s. a week, can, and usually does, pay for his medical treatment by joining a club; but for dental attentions he too often relies on the dental department of the local hospital. If this class, and the even more deserving clerk on £80 a year, can be reached, the scheme may surely be called philanthropic, whether or not it produces 5 per cent. interest. At any rate, it is one that should have the careful and earnest consideration of the profession.

